# Tree-ring isotopes adjacent to Lake Superior reveal cold winter anomalies for the Great Lakes region of North America

**DOI:** 10.1038/s41598-019-40907-w

**Published:** 2019-03-13

**Authors:** Steven L. Voelker, S. -Y. Simon Wang, Todd E. Dawson, John S. Roden, Christopher J. Still, Fred J. Longstaffe, Avner Ayalon

**Affiliations:** 1 Department of Plants, Soils and Climate, Utah State University, Logan, UT USA; 2 Ecology Center, Utah State University, Logan, UT USA; 3 Department of Integrative Biology, University of California – Berkeley, Berkeley, CA USA; 4 Department of Biology, Southern Oregon University, Ashland, OR USA; 5 Department of Forest Ecosystems and Society, Oregon State University, Corvallis, OR USA; 6 Department of Earth Sciences, Western University, London, Ontario Canada; 7 Geological Survey of Israel, Jerusalem, Israel

## Abstract

Tree-ring carbon isotope discrimination (Δ^13^C) and oxygen isotopes (δ^18^O) collected from white pine (*Pinus strobus*) trees adjacent to Lake Superior show potential to produce the first winter-specific paleoclimate reconstruction with inter-annual resolution for this region. Isotopic signatures from 1976 to 2015 were strongly linked to antecedent winter minimum temperatures (T_min_), Lake Superior peak ice cover, and regional to continental-scale atmospheric winter pressure variability including the North American Dipole. The immense thermal inertia of Lake Superior underlies the unique connection between winter conditions and tree-ring Δ^13^C and δ^18^O signals from the following growing season in trees located near the lake. By combining these signals, we demonstrate feasibility to reconstruct variability in T_min_, ice cover, and continental-scale atmospheric circulation patterns (r ≥ 0.65, P < 0.001).

## Introduction

Trees growing in cold environments do not directly record winter conditions in their tree-rings because they are dormant during this period. Isotopic signals imprinted upon tree-ring cellulose can serve as robust climate proxies that are conventionally known to record growing season conditions^1^. Indeed, the stable isotope composition of tree-ring cellulose has been shown to reflect canopy-integrated leaf responses to environmental drivers that are further modified by downstream ecophysiological processes^2–7^. More specifically, in temperate, near-coastal locations, the primary environmental signals recorded by tree-ring carbon and oxygen isotopes include warm season temperature, vapor pressure deficit, irradiance, and cloud cover^8–14^. Therefore, to our knowledge, no tree-ring signals of any type have been linked to winter temperatures or ice cover of lakes or oceans via well-understood meteorological and ecophysiological phenomena. To overcome this limitation, herein we first describe aspects of coastal “lake-effect” climate, and show how climate conditions adjacent to Lake Superior during spring and early summer are seasonally-lagged and driven by antecedent winter conditions. Thereafter we demonstrate how isotopic signals fixed in the central portion of each tree-ring appear to record winter temperature anomalies for the Great Lakes region of North America.

Lake Superior is the largest freshwater lake in the world by surface area and the third largest by volume. The resulting thermal mass produces “lake-effect” climate conditions that strongly regulate minimum and maximum air temperatures (T_min_ and T_max_, respectively) and other meteorological variables near the lake-land-atmosphere interface^15–19^. Ice forms around the shallow perimeter of Lake Superior every winter, but the lake surface rarely freezes over completely due to the large heat storage capacity of the lake^20–22^. Winter areal ice cover generally peaks near the first week of March at about 40%, but thin ice forms and melts earlier compared to lags of approximately two and four weeks for medium and thicker ice, respectively (Supplementary Fig. 1a). Inter-annual variation in peak ice cover across thickness classes is controlled primarily by winter minimum temperatures (Supplementary Fig. 1b).

Data from NOAA buoys deployed since 1979 reveal that Lake Superior water surface temperatures and over-lake air temperatures during summer are inversely related to ice coverage during the previous winter^23^ (Supplementary Fig. 1c). Lake-cooled air temperatures occur near the shoreline and at some distance inland, as demonstrated by spatial patterns of significant correlations between antecedent winter T_min_ and warm-season T_max_ during June, July and August (Supplementary Fig. 1d). The inland-advection of cool lake breezes is driven in part by greater overland warming and convection that can modify air temperature regimes up to 40 km from one of the Great Lakes^17,24–26^. Moreover, it is notable that inter-annual variability in surface water temperatures is amplified compared to regional air temperatures, corresponding to decreases in ice cover of 0.49% yr^−1^ over the same period (Supplementary Fig. 2). Amplified water temperature variability has been thought to represent ice albedo-effects^27^ interacting with the timing of stratification of lake water temperatures^23^.

Climate responses of tree-ring carbon and oxygen isotopes are widely acknowledged to record growing season conditions^1^, and this includes climate conditions along the US West Coast^10,28,29^. The climate of the US West Coast is influenced by ocean temperatures, but compared to Lake Superior, ocean temperature variability is muted and influenced primarily by coastal upwelling and large-scale atmospheric and ocean interactions. Due to the strong variability in Lake Superior water temperatures, we hypothesized that by sampling stable isotope signals fixed in cellulose during the spring and early summer, trees will have recorded winter season conditions rather than growing season conditions as have been demonstrated in other coastal locations. Here we test this hypothesis using tree-ring carbon and oxygen isotopes collected from white pines (*Pinus strobus* L.) growing 2.3 km distance from the Lake Superior shoreline, at a location where strong summer lake-effect air temperature gradients have been documented using a high-density network of temperature sensors^26^.

## Methods

**Figure 1:**
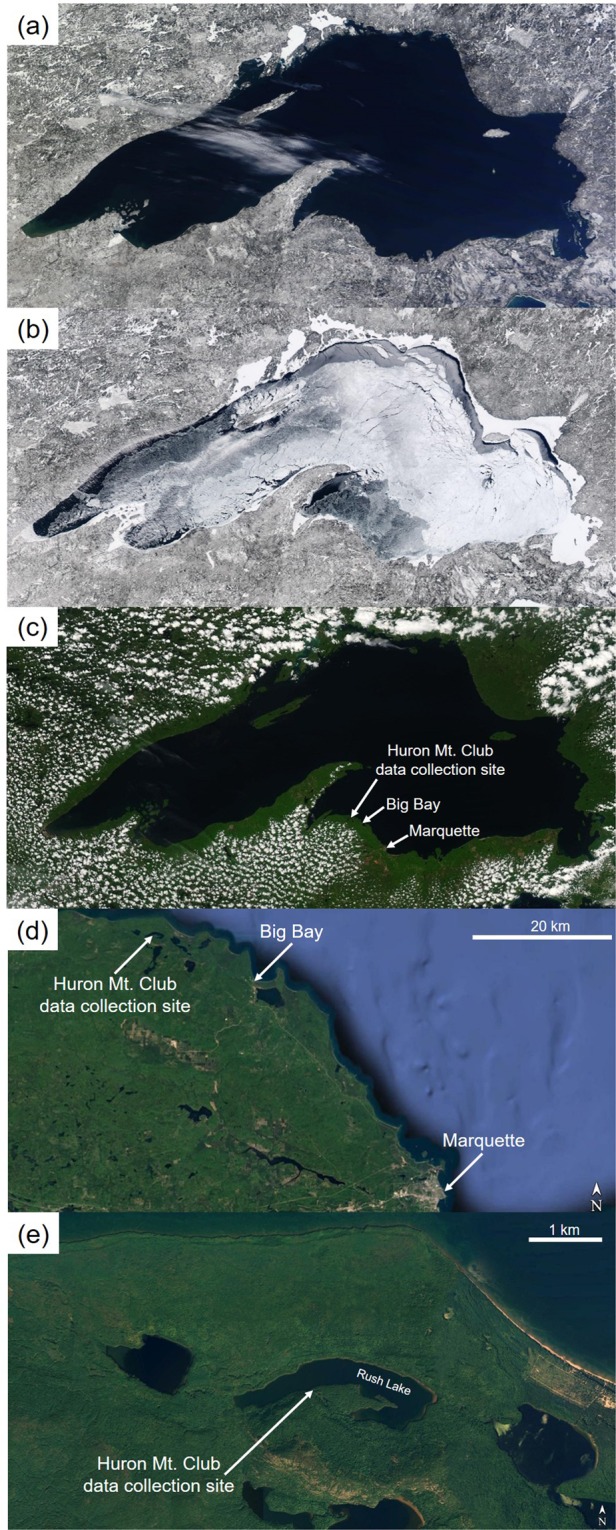
Images of variation in areal ice cover and cloud cover for the Lake Superior region noting the location of tree core collection and the nearest two meteorological stations. MODIS images, courtesy of the Space Science and Engineering Center (SSEC), University of Wisconsin-Madison and NASA (http://ge.ssec.wisc.edu/modis-today/), showing a year with extreme low winter ice cover on 3/11/2012 (**a**), extreme high winter ice cover on 3/11/2014 (**b**), and low cloud cover surrounding Lake Superior during the early growing season on 6/3/2010 (**c**). Higher resolution images (Map data: Google, Landsat/Copernicus) show the location of tree core collection on the south shoreline of Rush Lake, Michigan and the approximate locations of the nearest two meteorological stations at Big Bay and Marquette, Michigan (**d**,**e**).

We sampled five dominant or codominant white pine trees 2.3 km from the Lake Superior shoreline. Each tree was approximately 130 years in age and was located on the southern shoreline of Rush Lake, on the property of the Huron Mountain Club (Fig. 1). Three 12 mm-diameter cores were collected from each tree. Tree cores were mounted, surfaced and visually cross-dated. Thereafter rings were measured with a linear encoder (Velmex Inc., Bloomfield, NY) and MeasureJ2X software (Voortech Consulting) and statistically cross-dated against local and regional white pine chronologies (R. Fahey and S. Voelker, *unpublished data*) using COFECHA software^30,31^. After cross-dating, the middlewood^28^ (MW) and latewood components of each ring were excised with a Dremel tool and saved for analyses whereas the earliest cells formed in each ring were discarded (approximating 20% of the anatomically distinct earlywood). This practice minimized the potential for physiological carryover effects from previous growing season conditions^28^. Here, we only report data from the MW component of each ring – which should represent growth and isotope signals during late spring through early summer. After stable isotope analyses (described below), tree-ring isotope series from each tree were detrended with ARSTAN software^32^ using a 100-year spline. Detrending had the two-fold goals of (1) removing low frequency trends in the individual isotope time series that we expected were not due to climate, but which can result from changes in tree height, competition and rooting depth, and (2) retaining inter-annual to decadal-scale variation in the isotope time series that we expected to be related to inter-annual climate variability.

Isotopic composition is expressed using “delta” notation as δ^13^C or δ^18^O = (R_sample_/R_standard_ − 1) × 1000, where δ^13^C or δ^18^O is the molar ratio of heavy to light isotopes and R_standard_ is Vienna Pee Dee Belemnite (VPDB) or Vienna Standard Mean Ocean Water (VSMOW), respectively. Wood was ground to a powder, heat-sealed within a polyester filter bag (ANKOM Technology, Macedon, NY), extracted to α-cellulose^33,34^, and weighed out on a microbalance. Carbon isotope ratios were obtained using standard high temperature combustion in a vario-Pyrocube elemental analyzer interfaced with IsoPrime/Elementar IsoPrime 100 gas phase isotope ratio mass spectrometer (IsoPrime Ltd., Manchester, UK) at the Roden laboratory at Southern Oregon University. Oxygen isotope ratios were determined by pyrolizing α-cellulose in an elemental analyzer (TC/EA, IsoPrime/Elementar vario-Pyrocube) and analyzing the resulting gas with an isotope ratio mass spectrometer (IsoPrime100) at the Dawson laboratory at UC-Berkeley. At these laboratories, the long-term precision is less than 0.1‰ for δ^13^C and 0.2‰ for δ^18^O. δ^13^C data were converted to Δ^13^C following Farquhar (1989) as: Δ^13^C = (δ^13^C_air_ − δ^13^C_*plant*_)/(1 + δ^13^C_*plant*_/1000) where δ^13^C_air_ was estimated from McCarroll and Loader (2004) through 2003 and merged seamlessly with a similarly smoothed record of annual δ^13^C_air_ from Mauna Loa, Hawaii (http://scrippsco2.ucsd.edu/data/atmospheric_co2/mlo) for 2004 to 2015.

Daily resolution temperature data, from buoys and meteorological stations near the lake were from the United States National Ocean and Atmospheric Association National Buoy Data Center (http://www.ndbc.noaa.gov/maps/WestGL.shtml) and the Canadian Government (http://climate.weather.gc.ca/). Daily resolution cloud cover data were from the NOAA Great Lakes Environmental Research Laboratory (https://www.glerl.noaa.gov/). The Big Bay and Marquette, MI meteorological stations are 15 and 55 km to the southwest of the tree-ring collection site, respectively. Both stations are located <0.1 km from Lake Superior. After summarizing temperature data by month at each site, missing and/or incomplete monthly values for either record were gap-filled using linear regressions developed between the two sites. Hereafter we define this site-averaged data set to be the “local” temperature signal. We also summarized meteorological data from all stations within 7 km of the Lake Superior, which we here define as the “regional near-lake” mean. Likewise, regional near-lake cloud cover data were summarized from airports within 7 km of Lake Superior.

For regional and local winter T_min_, we averaged monthly values across the previous November to the current March to provide a winter seasonal T_min_ whereas summer seasonal data were averaged across the current June to August. One potential concern of using temperature data from lake-effect locations is that summer T_max_ is significantly (P < 0.01) influenced by winter T_min_, whereas upwind locations that are outside of the influence of Lake Superior do not show these patterns (Supplementary Fig. 1d). To remove covariation among these signals, we obtained residuals, from a linear regression relating summer T_max_ to winter T_min_ using data summarized from Marquette and Big Bay (*data not shown*), to produce a warm-season temperature record independent of the effect of winter T_min_, which are hereafter referred to as T_max_*.

Weekly ice cover estimates were obtained from the Canadian Ice Service (http://iceweb1.cis.ec.gc.ca/IceGraph/page1.xhtml). Thin, medium, and thick ice cover were averaged into a single value per year across weeks 7–12, 9–14 and 11–16, respectively, with the week of January 1^st^ defined as week zero. This procedure combines ice cover signals while accounting for lags in the formation of medium and thick ice (Supplementary Fig. 1a).

Winter cold outbreaks across the Midwest, southern Canada and Eastern United States have been associated with low atmospheric pressure or geopotential height (HGT) anomalies, generally centered between Lake Superior and the eastern edge of Hudson Bay, Canada^35–37^. The fluctuation of such winter “stationary waves” in the upper atmosphere modulates surface air temperature across the Great Lakes Region. Indeed, the “Polar Vortex” event of the 2013–14 winter easily set a new record of areal ice cover on Lake Superior since records have been kept. This included a peak coverage of 100%, with some ice remaining into early June visible from MODIS images (Fig. 1a). The winter climate over North America and particularly the Great Lakes is affected by mean-state stationary atmospheric waves, characterized by a pair of circulation features in the upper troposphere: A high-pressure ridge over western North America and a low-pressure trough over the Hudson Bay area^38^, referred to as the dipole^36,37^. To characterize this continental-scale circulation pattern, we obtained HGT anomaly data at 250 hPa from the two centers of this dipole: over the Northeastern Pacific Ocean and the eastern edge of Hudson Bay, Canada, respectively. The difference between these anomalies has been called the “dipole index”^35,36,39^ and the HGT anomaly values forming this dipole index were derived from data with a 2.5° latitude × longitude resolution from the National Centers for Environmental Prediction/National Center for Atmospheric Research Reanalysis^40^. Linear regression and multiple linear regression models were constructed and compared using the “lm” or “glm” packages in the R statistical computing environment version 3.4.3^41^.

## Results

Winter T_min_, the strongest driver of ice formation on Lake Superior, differs greatly from year to year, as demonstrated by MODIS images from early March that contrast ice conditions during the relatively warm winter of 2011–2012 (Fig. 1a) versus the cold and so-called “Polar Vortex” winter of 2013–2014 (Fig. 1b). In spring and summer, the thermal inertia of Lake Superior causes the lake surface to be much cooler than adjacent land surfaces, resulting in subsidence of air passing over the lake and advection of cool lake breezes inland while stabilizing the surrounding atmosphere. These effects can be visualized collectively through a snapshot of cloud cover surrounding the perimeter of Lake Superior, but essentially no cloud cover over the lake (Fig. 1c).

White pine tree-ring δ^18^O and Δ^13^C chronologies were characterized by series inter-correlation of 0.76 and 0.54, respectively, while expressed population signals^42^ were 0.94, and 0.85, respectively. These characteristics are a hallmark of chronologies that have a robust common signal driven by climatic variability. To determine the strongest climate drivers of Δ^13^C and δ^18^O, multiple regression analyses were conducted that included local (winter T_min_ and summer T_max_* from the two closest meteorological stations), regional (summer cloud cover and lake-wide peak ice cover %,), and large-scale variables (winter HGT and winter dipole index) for the current year (Table 1).

**Table 1:** Results from multiple regression analyses predicting inter-annual variation in Δ^13^C, δ^18^O from current year climate data.

Variable	Model	Adjusted R^2^	Model vs predictand correlation	P-value
Δ^13^C	Full model	0.26	0.63	**0**.**0169**
Δ^13^C	Ice cover + HGT	0.33	0.62	**0**.**0006**
δ^18^O	Full model	0.30	0.65	**0**.**0082**
δ^18^O	Winter T_min_ + Dipole Index	0.30	0.58	**0**.**0006**

The “Full model” includes all terms; Winter T_min_, Summer T_max_* Near-lake Winter T_min_, Near-lake cloud cover, Peak ice cover, Geopotential height (HGT) and Dipole index. Results from an alternative model, retaining only significant (P < 0.05) predictor variables are also listed. For models considering more than one predictor variable the absolute value of model vs predictand correlations are given. See Methods for definitions of variables.

In contrast to most other studies of tree-ring temperature signals, only winter season variables were found to be significant and thereafter retained in the final multiple regression models for white pine MW Δ^13^C and δ^18^O (Table 1). Ice cover and local winter T_min_ values had the strongest influence on the MW Δ^13^C and δ^18^O, respectively, whereas HGT and dipole index also described significant variation in the same models (Table 1). The modeling process was then inverted, with the goal of identifying targets for climate reconstruction utilizing MW Δ^13^C and δ^18^O.

**Figure 2:**
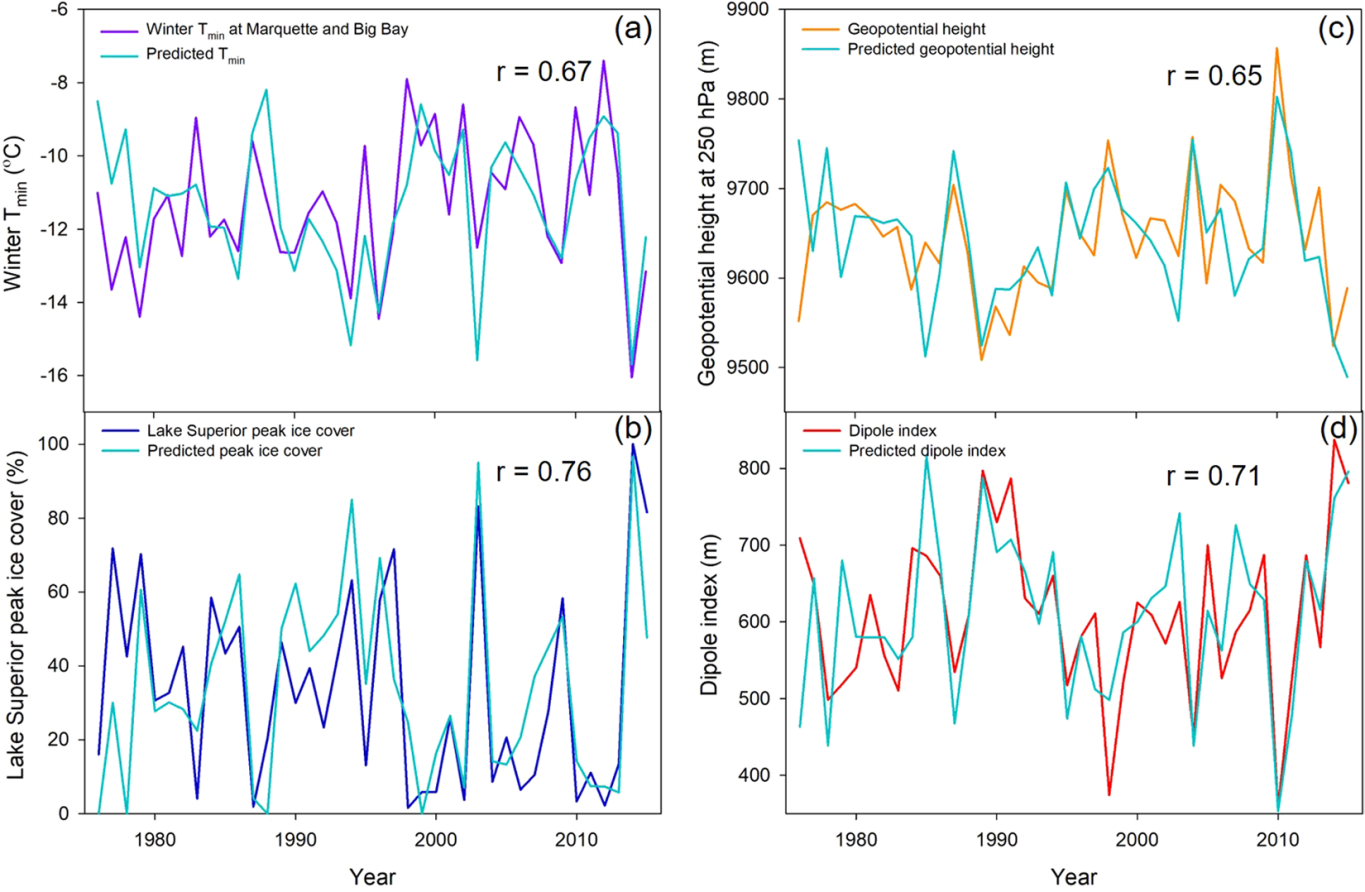
White pine Δ^13^C & δ^18^O chronologies collected at the Huron Mt. Club, Michigan used to predict inter-annual variation in winter minimum temperatures (T_min_) (**a**), Lake Superior peak ice cover (**b**), regional geopotential height (HGT) centered near Hudson Bay, Canada (**c**) and the dipole index defining differences between HGT centers over the Northeast Pacific Ocean and Hudson Bay (**d**). Winter T_min_ represents the mean values from Big Bay and Marquette, Michigan averaged across the previous November to the current March and reconstructed values. As in Table 2, current and subsequent year Δ^13^C & δ^18^O values were used to predict each variable for each year except for 2015, in which only current year isotope data were available.

All winter-season variables were strongly predicted by individual isotope chronologies and their dual-isotope combinations (Fig. 2). For the variables included in climate reconstructions (Fig. 2), variance inflation factors were low, <2, indicating that multicollinearity did not contribute to over-fitting the model. Across all variables there were no significant relationships with summer T_max_* or summer near-lake cloud cover (Table 2). It is notable for interpreting these results that summer T_max_* is the summer temperature record after having removed the impact of winter T_min_ due to the lagging response of lake-effect climate (See Methods). This means that stable isotope variation did not reflect variability in regional summer climate conditions that were independent of from lake-effect climate variability. The strength of the relationships noted in Table 2 and that between ice cover and the dipole index increased only weakly through time (Supplementary Fig. 3). These results clearly indicated that, for this species, spring and early summer conditions near the lakeshore were dominated by the influence of winter season climate signals.

**Table 2:** Inter-annual variation in seasonal climate variables predicted by inter-annual variation in Δ^13^C, δ^18^O in linear regression analyses or both isotope signals in multiple regression analyses.

Spatial scale	Variable	Model	Adjusted R^2^	Model vs predictand correlation	P-value
Local	Winter T_min_	Δ^13^C	0.23	0.52	**0**.**0034**
Local	Winter T_min_	δ^18^O	0.16	0.45	**0**.**0075**
Local	Winter T_min_	Δ^13^C & δ^18^O	0.38	0.67	**0**.**0004**
Local	Summer T_max_*	Δ^13^C	0.01	0.24	0.3308
Local	Summer T_max_*	δ^18^O	0.00	0.22	0.4185
Local	Summer T_max_*	Δ^13^C & δ^18^O	0.00	0.31	0.4726
Regional	Near-lake Winter T_min_	Δ^13^C	0.29	0.57	**0**.**0007**
Regional	Near-lake Winter T_min_	δ^18^O	0.11	0.39	**0**.**0479**
Regional	Near-lake Winter T_min_	Δ^13^C & δ^18^O	0.32	0.68	**0**.**0002**
Regional	Near-lake cloud cover	Δ^13^C	0.00	0.14	0.7178
Regional	Near-lake cloud cover	δ^18^O	0.06	0.33	0.1272
Regional	Near-lake cloud cover	Δ^13^C & δ^18^O	0.04	0.37	0.2663
Regional	Peak ice cover	Δ^13^C	0.39	0.65	**<0**.**0001**
Regional	Peak ice cover	δ^18^O	0.14	0.43	**0**.**0265**
Regional	Peak ice cover	Δ^13^C & δ^18^O	0.53	0.76	**<0**.**0001**
Large-scale	HGT	Δ^13^C	0.22	0.51	**0**.**0046**
Large-scale	HGT	δ^18^O	0.11	0.40	**0**.**0446**
Large-scale	HGT	Δ^13^C & δ^18^O	0.35	0.65	**0**.**0008**
Large-scale	Dipole index	Δ^13^C	0.25	0.54	**0**.**002**
Large-scale	Dipole index	δ^18^O	0.16	0.45	**0**.**0169**
Large-scale	Dipole index	Δ^13^C & δ^18^O	0.44	0.71	**<0**.**0001**

Variables are ordered by spatial scale classes from top to bottom. P-values in boldface type are significant at P < 0.05. All model results represent the climate variable predicted by isotope values of the current and subsequent year to address autocorrelation in climate variables related to the thermal inertia of Lake Superior. See Table S1 for results exclusively from current year isotope signals. The absolute value of model vs predictand correlations are given for all regression models. Geopotential height is abbreviated as HGT, see Methods for definitions of variables.

Compared to models using current and antecedent year isotopic signals (Table 2), when only current year signals were considered, climate variables that operate at local to regional scales, such as winter T_min_ and ice cover, were predicted somewhat better compared to larger-scale influences on winter atmospheric circulation, such as winter HGT and the winter dipole index (Supplementary Table 1). Current year Δ^13^C predicted variability in all of the winter climate signals reasonably well, with little additional explanatory power provided by current year δ^18^O for either of the winter HGT or dipole index (Supplementary Table 1). However, predictions of T_min_ and peak ice cover were substantially improved by including Δ^13^C of the subsequent year, and predictions of HGT and the winter dipole index improved substantially by including δ^18^O of the subsequent year (cf. Table 2 and Supplementary Table 1). Atmospheric pressure patterns and attendant “waviness” of the jet stream produces winter weather conditions at a given location but these large scale phenomena do not directly affect isotopic signatures. Likewise, ice cover on Lake Superior is largely a product of winter T_min_ (Supplementary Fig. 1b). Therefore, ultimately, winter temperatures drove tree-ring isotopic variation over time, with spatial signatures of this relationship being strongest over south-central Canada, the upper peninsula of Michigan and Northern Wisconsin (Supplementary Fig. 4).

**Figure 3:**
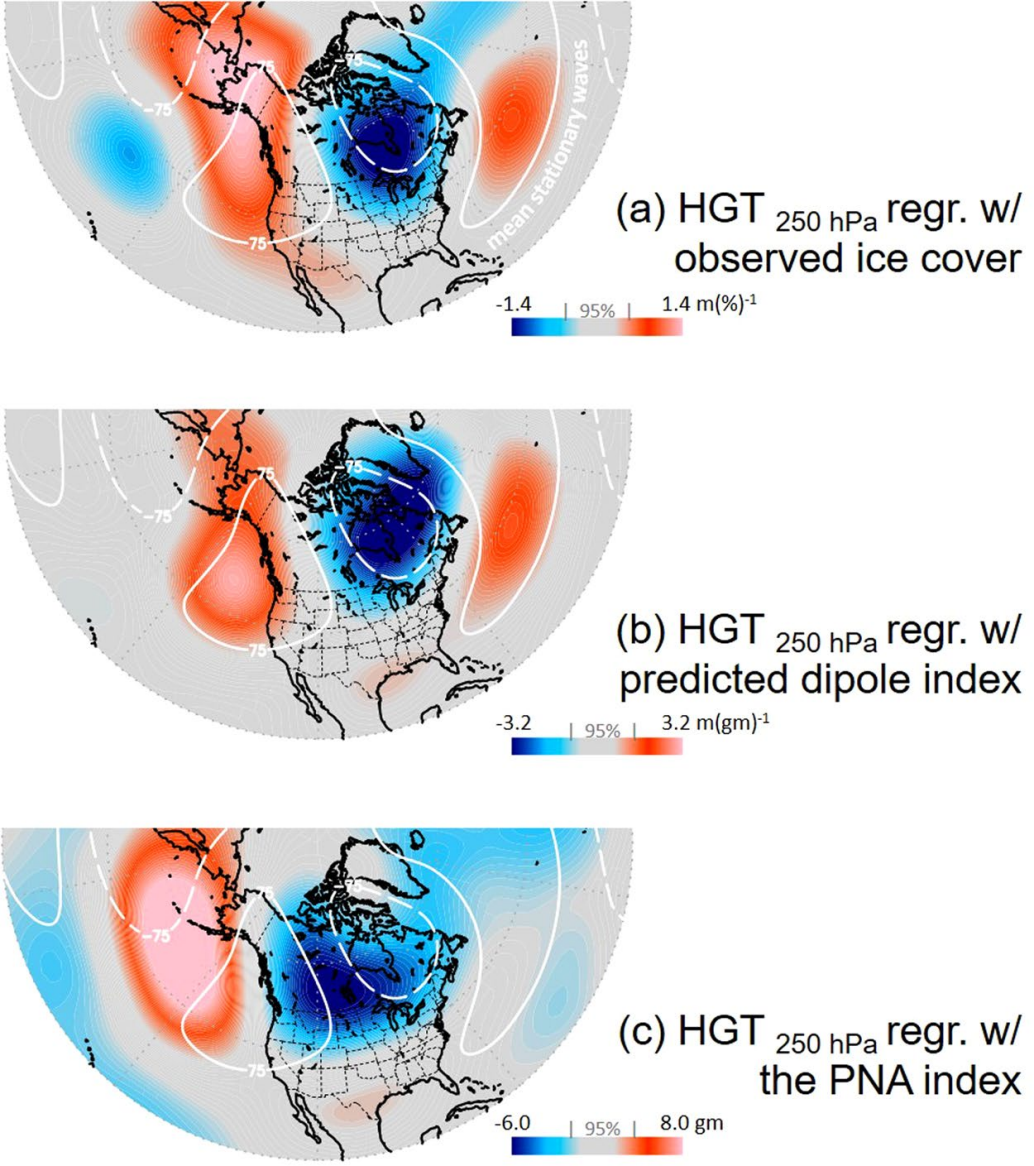
Anomalous patterns of winter (Nov-Feb) 250 hPa eddy geopotential height (HGT, without the zonal mean) regressed against the observed peak ice cover for Lake Superior from Fig. 2b (**a**), the predicted dipole index from Fig. 2d (**b**), and the PNA index obtained from the Climate Prediction Center/NOAA, as shadings (**c**). The winter-mean stationary wave eddies are overlaid in all three panels as white contours, constructed from the 1981–2010 climatology.

A regression map of the peak ice cover regressed with the 250 hPa geopotential height eddies (HGT, i.e. with the zonal mean removed for the depiction of stationary waves in the upper atmosphere) elucidates large-scale circulation patterns producing cold anomalies and associated inter-annual variability in ice cover (Fig. 3a). As expected, an east-west oriented wave-train pattern is revealed over North America that spans the North Pacific to North Atlantic. For comparative purposes, the climatological stationary waves are overlaid as contours, outlining the “normal” position of the stationary eddies during winter (Fig. 3a). Over North America, the regression pattern of the circulation associated with the inter-annual variation in ice cover corresponds closely to the winter-mean stationary wave patterns, signaling that amplified stationary waves produce cold anomalies across the northeast U.S. and southern Canada^36,39,43^ and associated increases in ice cover for Lake Superior. Similarly, in Fig. 3b, the regression map of HGT with the dipole index predicted from tree-ring Δ^13^C and δ^18^O (i.e., from Fig. 2d) also shows a dipole pattern, despite a slight difference in the ridge intensity over Alaska. In contrast to the similar dipole patterns in Fig. 3a,b, the regression map of HGT with the well-known Pacific-North America pattern (PNA)^44^ shows a distinctly different and spatially shifted pattern compared to the dipole position. As a result, tree-ring Δ^13^C and δ^18^O chronologies and peak ice cover were not significantly correlated with the PNA (*data not shown*).

## Discussion

Our results provide evidence that tree-ring Δ^13^C and δ^18^O of white pines growing adjacent to Lake Superior show potential to provide novel proxy records of winter temperature anomalies with inter-annual resolution. Although we sampled only five trees at one location, the resulting tree-ring Δ^13^C and δ^18^O chronologies were able to predict previous winter temperature and ice conditions locally and regionally, as well as depicting the dominant large-scale atmospheric circulation pattern associated with these winter temperature and ice conditions (Figs 2, 3; Table 2). There are limitations to using a data set from only one site, since climatic controls on tree-ring isotopic variation can differ depending on microsite conditions, and specifically with distance to water bodies^45^. It is possible that the sampling location was more responsive to lake effect climate than others at similar distances from Lake Superior or that adding additional sites may further strengthen winter climate signals affecting this region. Additional research would logically address whether comparable winter climate-isotope signals can be obtained from other locations around Lake Superior, to what extent distance from Lake Superior or variation in local geomorphology can modify these signals. Toward this end, tree-ring isotope carbon chronologies from other species from this region have demonstrated that upland sites with relatively thin soils overlying bedrock tend to more closely reflect the degree of summer drought stress (S. Voelker, *unpublished data*). Therefore, to obtain coherent winter climate signals from tree-ring isotopes it will likely be critical to sample trees from mesic but well-drained sites, which often occur next to lakes. Sampling trees from such sites has the added benefit of the potential to extend chronologies back in time by locating sub-fossil wood preserved in lakes that grew under similar climate and hydrologic conditions^46^. Finally, at any given site, tree ring-width and stable isotope records may not display long-term stability with climate^47^, so it will be important to extend isotope chronologies further back in time to ascertain this possibility over the full instrumental climate record. Despite the importance of sampling considerations for future studies aimed at exploiting the unique lake-effect climate signals demonstrated here, it is important to first address which physiological and climatic processes best explain how tree-ring carbon and oxygen isotopic signals record antecedent winter temperatures in our current data set.

According to carbon isotope theory, Δ^13^C is primarily controlled by the ratio of photosynthetic assimilation rate to stomatal conductance (*A/g*_s_)^48^, which results in two physiological processes which can be modified by climate variability. Tree-ring Δ^13^C is often strongly influenced by *g*_s_, as indicated by tree-ring Δ^13^C relationships with soil moisture deficits and/or atmospheric drought stress^14,48–51^. However, *g*_s_ is unlikely to constrain Δ^13^C near Lake Superior during spring and early summer because the air is cool and humid, the soil should still be wet from snowmelt, and precipitation >5 mm occurs on about 50% of days. Indeed, monthly Palmer Drought Severity Index showed no significant relationships with MW Δ^13^C (Supplementary Table 2). Theory relating Δ^13^C to *A/g*_s_^45^ suggests the primary remaining variable that could potentially explain inter-annual variation in MW Δ^13^C, is how late spring and early summer air temperatures affect *A*, as influenced by antecedent winter conditions. Near Marquette, average air temperatures during June and July include a T_min_ of 11.8 °C and T_max_ of 22.4 °C. *A* responds positively across this range of air temperatures, peaking near 20 °C for most temperate species^52,53^. Therefore, close to the lake, Δ^13^C signals from early in the growing season are fixed in the MW component of tree-rings in association with frigid previous winters. This is caused by near-lake temperatures during this period being inversely proportional to ice cover such that *A* is constrained by cooler T_max_ adjacent to the lake^26^ and more strongly so after winters with colder T_min_.

Tree-ring δ^18^O is primarily influenced by source water isotopic signatures and the degree to which leaf water is either enriched by evaporation or influenced by atmospheric water vapor^2–4,14,51^. Source water isotopic signals near Lake Superior surely contributed to some of the inter-annual variation in the tree-ring δ^18^O patterns observed due to how temperatures influence precipitation δ^18^O^54–56^. Monthly temperatures can explain about 67% of the variation in monthly-resolution precipitation δ^18^O data from 1995 to 2015 located 180 km North across Lake Superior from our collection site at Sibley, Ontario (F. Longstaffe, *unpublished data*). White pine MW δ^18^O also had a strong relationship (R^2^ = 0.47, P < 0.001) with the same Sibley precipitation δ^18^O data centered on the late winter to early spring period over the previous two years (Supplementary Fig. 5). Finally, Sibley precipitation δ^18^O averaged across the previous November to current March was significantly correlated with HGT and the Dipole index (r > 0.49, P < 0.027, *data not shown*), thereby linking winter atmospheric circulation, precipitation δ^18^O and tree-ring δ^18^O. The apparent integration of late winter precipitation δ^18^O signals across two previous years rather than just one likely reflects the time it takes for winter snow to melt and move down the slope of the north face of Huron Mountain, to the base of the mountain where seeps and springs commonly occur along the shoreline of Rush Lake near where the white pine trees were sampled for this study. Hence, this link to conditions across two previous winters for tree-ring δ^18^O may not be as strong at sites with less varied local topography. On the other hand, at other sites that lack a strong influence of lateral soil water flow, we would expect the current year precipitation δ^18^O signals to have a relatively stronger influence on tree-ring δ^18^O.

Lake-effect precipitation is known to affect large spatial gradients in water isotopes, across distances of 50 kilometers or more in lower Michigan^57^. However, there is only a very weak relationship between winter T_min_ and winter precipitation amounts from data compiled across all near-lake stations including the years 1900–2015 (R^2^ = 0.02, P = 0.1574) or across only the Marquette and Big Bay locations including the years 1966–2015 (R^2^ = 0.01, P = 0.4114). Tree-ring δ^18^O also had very weak correlations with winter precipitation records from all near-lake stations or from Marquette and Big Bay (r = −0.11 and 0.06, respectively, *data not shown*) and summer precipitation (r = −0.16 and −0.20, respectively, *data not shown*). Monthly Palmer Drought Severity Index also showed no significant relationships with MW δ^18^O (Supplementary Table 2). The PNA pattern contributes to continent-wide patterns in precipitation δ^18^O from the Northwest to Southeast across central North America, but interpolations of isotopic variation suggest there is no difference in precipitation δ^18^O between PNA phases for Lake Superior and the upper Great Lakes Region^58^.

Another influence on inter-annual variation in tree-ring δ^18^O surely derives from leaf water δ^18^O dynamics during the spring and early summer. Indeed, the dominant control over tree-ring δ^18^O was air temperatures during the early growing season, which were in turn strongly influenced by antecedent winter T_min_ and other unmeasured winter conditions producing variation in winter ice cover (Table 2). This pattern can largely be attributed to a reduction in leaf temperature and leaf water evaporative enrichment by cold and humid air adjacent to the lake, particularly under conditions where lake breezes advect cool air inland. Overall, tree-ring δ^18^O formed during the spring and early summer is influenced by winter temperatures via source water (late winter precipitation δ^18^O) and indirectly via leaf water enrichment (lake-effect lags in lake temperature due to previous winter conditions). In contrast, the amount of lake-effect precipitation or phases of the PNA appear to have had little influence on tree-ring δ^18^O.

Agreement between winter-mean stationary wave eddies and the wave-train patterns produced by regression maps of HGT with Lake Superior ice cover and the dipole index predicted from tree-ring Δ^13^C and δ^18^O (Fig. 3a,b) contrasts sharply with long-term mean atmospheric circulation patterns associated with the PNA pattern (Fig. 3c). Physically, this translates to the dipole pattern reflecting amplification or attenuation of the mean stationary atmospheric wave pattern^59^, whereas the PNA describes atmospheric circulation patterns that are comparatively shifted in space.

The potential for using tree-ring isotopes to better understand past variation in winter conditions is strongly evident from the data and analyses presented here. Both carbon and oxygen isotopic signals fixed in the MW of white pines reflected winter conditions through the lagging lake-effect climate near the shore of Lake Superior interacting with well-known ecophsyiological drivers of how trees and leaves record environmental influences. Hence, there appears to be strong potential for reconstructing winter temperatures or atmospheric circulation patterns influencing this region using an intra-annual tree-ring sampling and a dual isotope approach. These same climate signals are also highly correlated with winter ice cover for Lake Superior, which raises the possibility of better understanding past ice dynamics that are a major determinant of annual evaporation and associated lake levels for Lake Superior^60^ and can also affect the limnological metabolism of this important ecosystem^61^.

## Supplementary information

Supplementary Information
