# Supplementary material for: Tree-ring isotopes adjacent to Lake Superior reveal cold winter anomalies for the Great Lakes region of North America
Source: Sci Rep. 2019 Mar 13;9:4412. doi: 10.1038/s41598-019-40907-w (PMC6416397; doi:10.1038/s41598-019-40907-w)
Supplement: Supplementary file 1 — Supplementary Information [file 41598_2019_40907_MOESM1_ESM.docx]

**Supplementary Information for: “Tree-ring isotopes adjacent to Lake Superior reveal cold winter anomalies for the Great Lakes region of North America”**

A manuscript submitted to *Scientific Reports*

Authors: Steven L. Voelker, Shih-Yu Simon Wang, Todd E. Dawson, John S. Roden, Christopher J Still, Fred J. Longstaffe and Avner Ayalon

**Figure 1.** Selected aspects of ice and air and water temperature dynamics for Lake Superior and areas nearby. Average timing of the formation of thin, medium and thick ice on Lake Superior (a). Relationship of peak ice cover versus winter minimum air temperature average across the previous November to current March (b). Mean Lake Superior spring/summer water surface and air temperatures measured at three NOAA buoys, plotted versus ice cover of the previous winter (c). Correlations of monthly maximum temperatures (T_max_, mean of June to August) versus previous winter minimum temperatures (T_min_, mean of previous November to current March) from 1966 to 2015 (d). The red horizontal line gives the correlation value above which these climate signals are significantly correlated at P < 0.01. Note that the Marquette and Big Bay record, as well as the regional near-lake mean stations (i.e. within 7 km of the lakeshore) display significant correlations between monthly summer T_max_ and previous winter T_min_ whereas stations from areas upwind and outside of the lake effect zone in Minnesota display no such significant correlations. This evidence demonstrates how summer temperatures near Lake Superior are uniquely linked to previous winter temperatures through lake-effect climate dynamics. The bars labelled as Minnesota represents the average correlations across three upwind locations (St. Paul, Grand Rapids and International Falls) that are outside of the influence of Lake Superior.


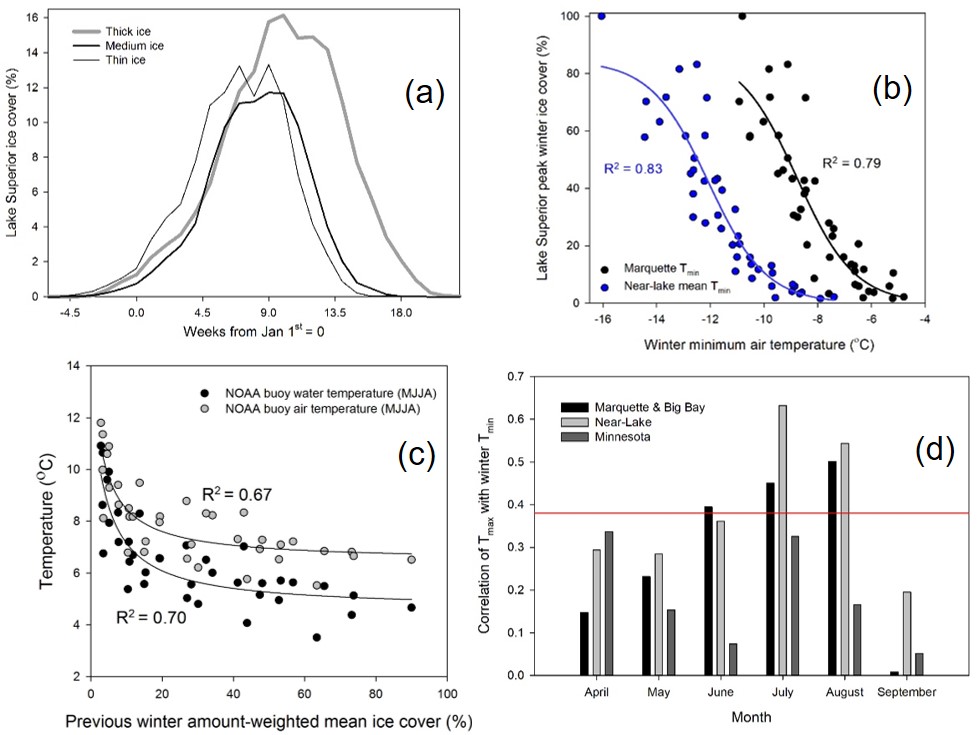


**Figure 2.** Upper panel: Trends in lake water surface temperatures measured from three buoys moored in Lake Superior and air temperatures from thee upwind locations (International Falls, Grand Rapids and Minneapolis, MN). Each data set was standardized by subtracting the value in 1979. Regional mean annual temperatures have risen 0.031 ºC yr^-1^, faster than the July air temperatures, but still more than two-fold slower than July water temperatures. For reference, summer (May-August) water temperatures have increased 0.051 ºC yr^-1^ (*data not shown*). Lower Panel: Lake Superior peak areal ice cover percentage from the previous winter (e.g. data plotted for 2017 correspond to the winter of 2016-2017).


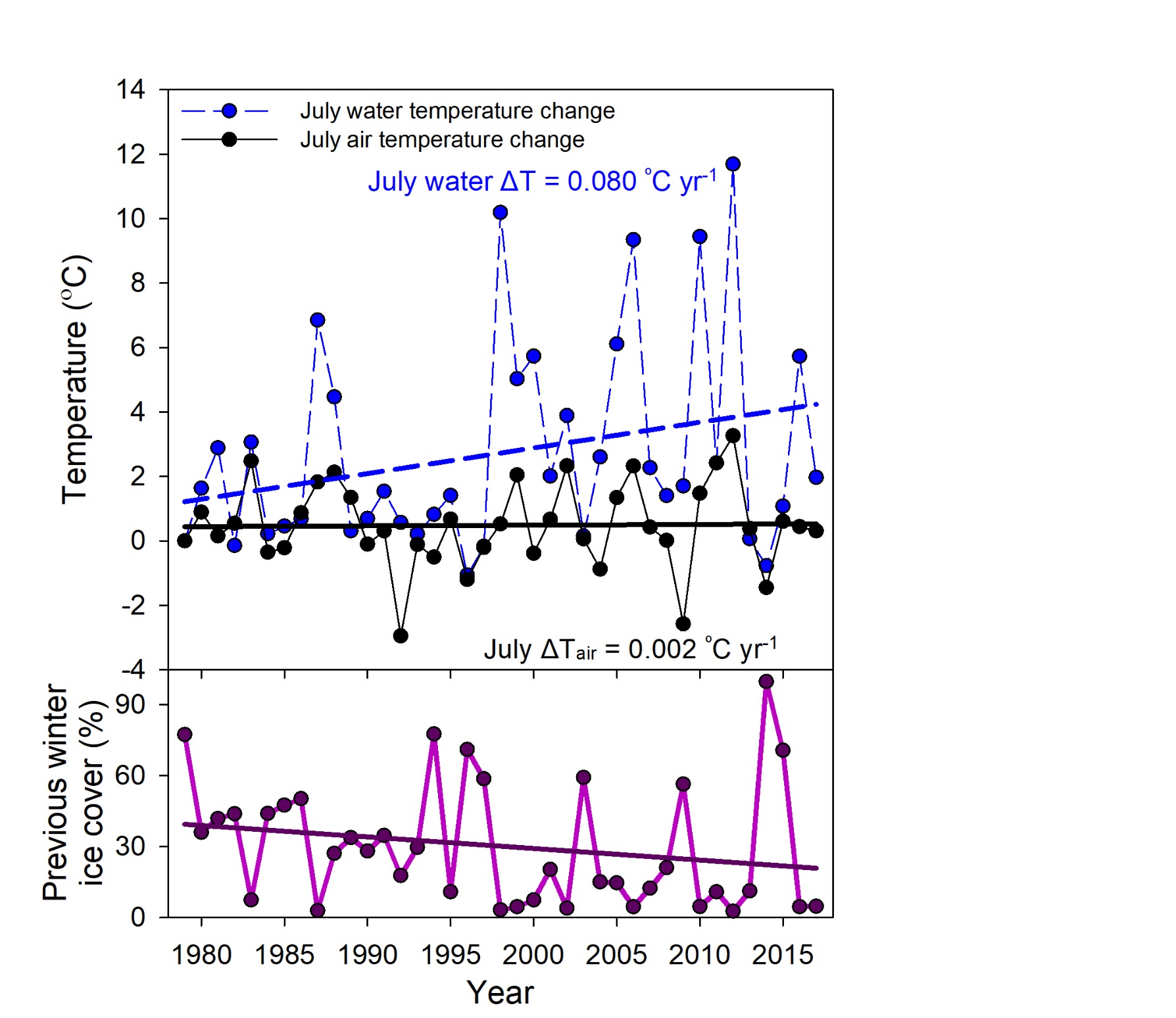


**Figure 3.** 11-year moving window correlation (r) values plotted over time for winter minimum temperatures, ice cover, geopotential height and the dipole index displayed in Figure 2. There are weak trends toward greater correlations during more recent years for most variables. Indeed, some degree of changes in variation explained would be expected if the dipole index has become more variable over the past 50—100 years (Wang et al. 2015). Changing variation in a climate or meteorological variable does not preclude paleo-reconstructions unless there is a drastic change whereby the signal strength is no longer detectable or opposite in sign. A longer tree-ring isotope data set extending across the instrumental period would allow for a more robust characterization of signal stability and how that may scale with variation in the dipole index.


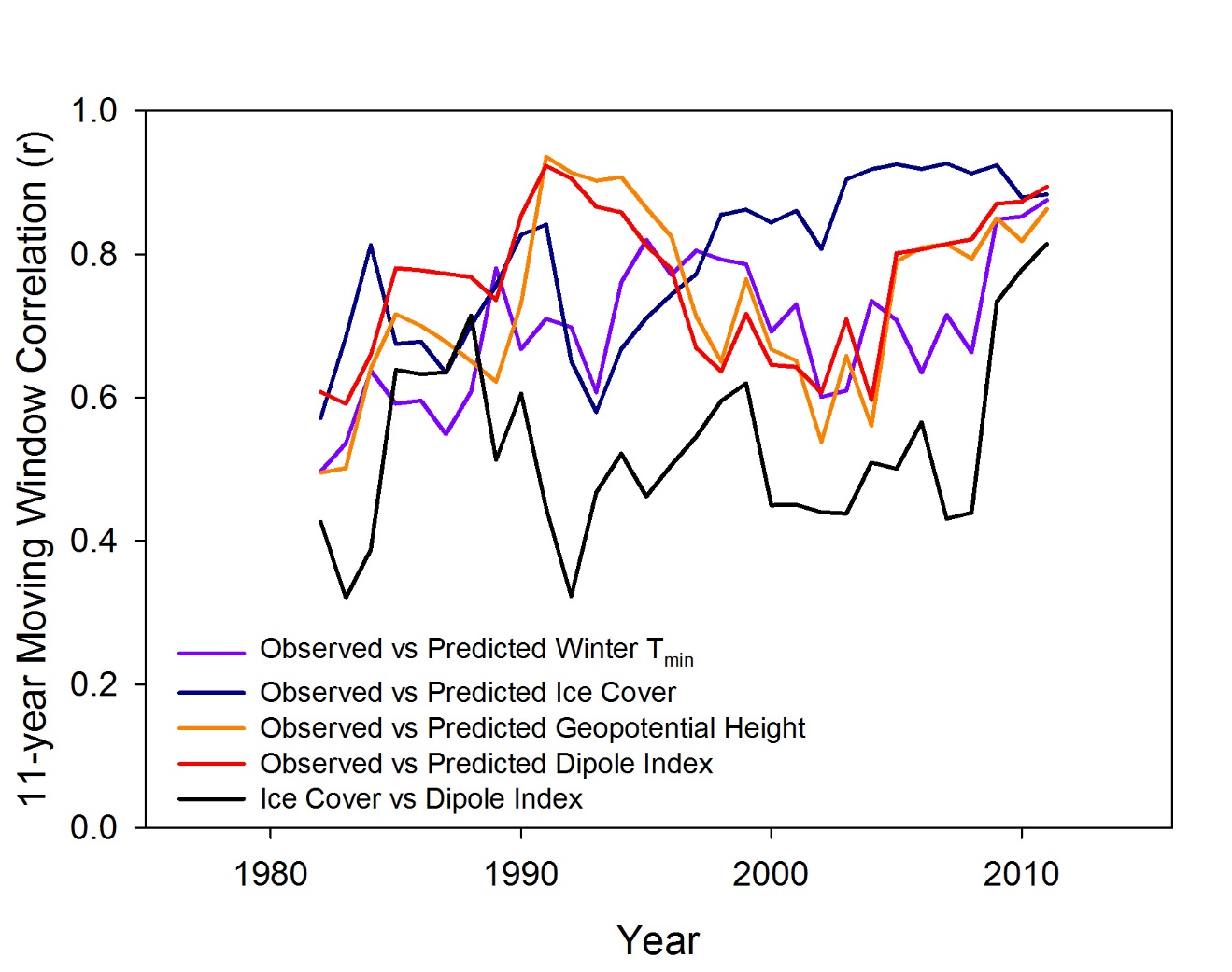


**Figure 4.** Correlation map analysis between the dipole index and 0.5 degree gridded CRU surface temperature data during December to February (DJF) over the 1985-2017 (Top panel) and 1948-1984 periods, with significant correlations (P < 0.05) covering much of the Great Lakes region. These results signify that the tree-ring Δ^13^C and δ^18^O series, given their high correlation with the dipole, reflects and can therefore depict the regional temperature variability across periods. The earlier period shows slightly weaker relationships, in line with weaker variability in the dipole index shown by Wang et al. (2017). More critically, the spatial pattern of the dipole influence on surface air temperature, as well as the significance region encompassing our sites, has not changed.


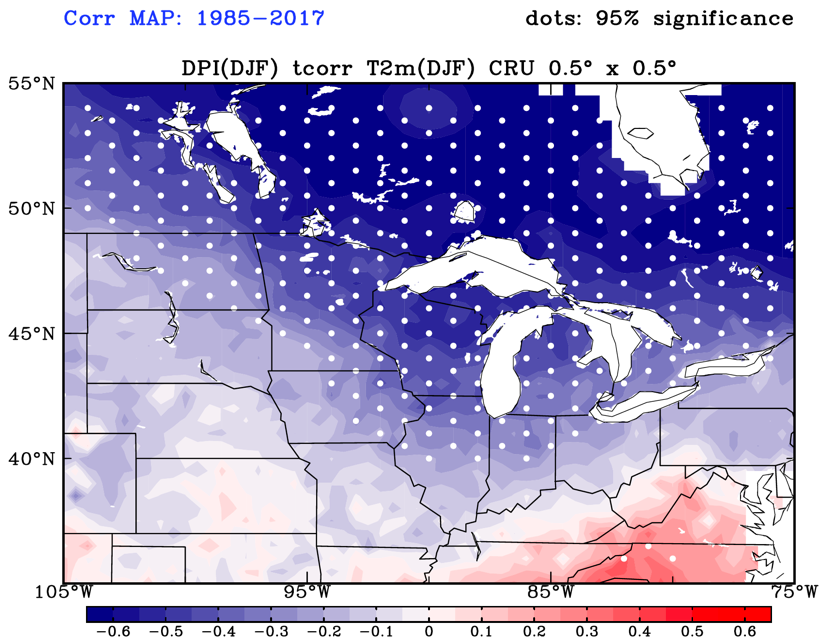


**
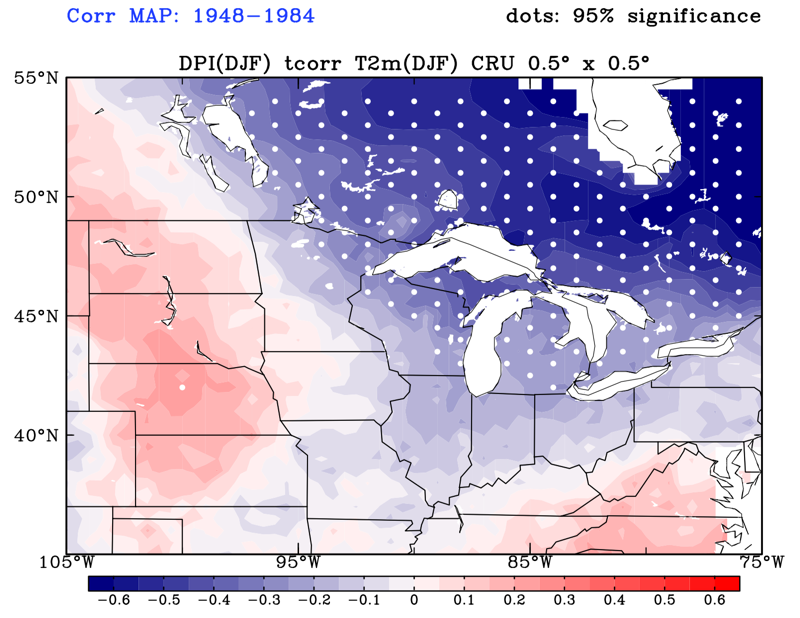
**

**Figure 5.** A plot and regression analysis of white pine middlewood cellulose δ^18^O, after detrending with ARSTAN, plotted against monthly precipitation δ^18^O data relative to the VSMOW standard (F. Longstaffe, *unpublished data*). Precipitation δ^18^O data were averaged across February to May of the current and previous year. There were also significant relationships, albeit somewhat weaker, with precipitation δ^18^O averaged across the water year or just for winter months, but the relationship shown below was the strongest.


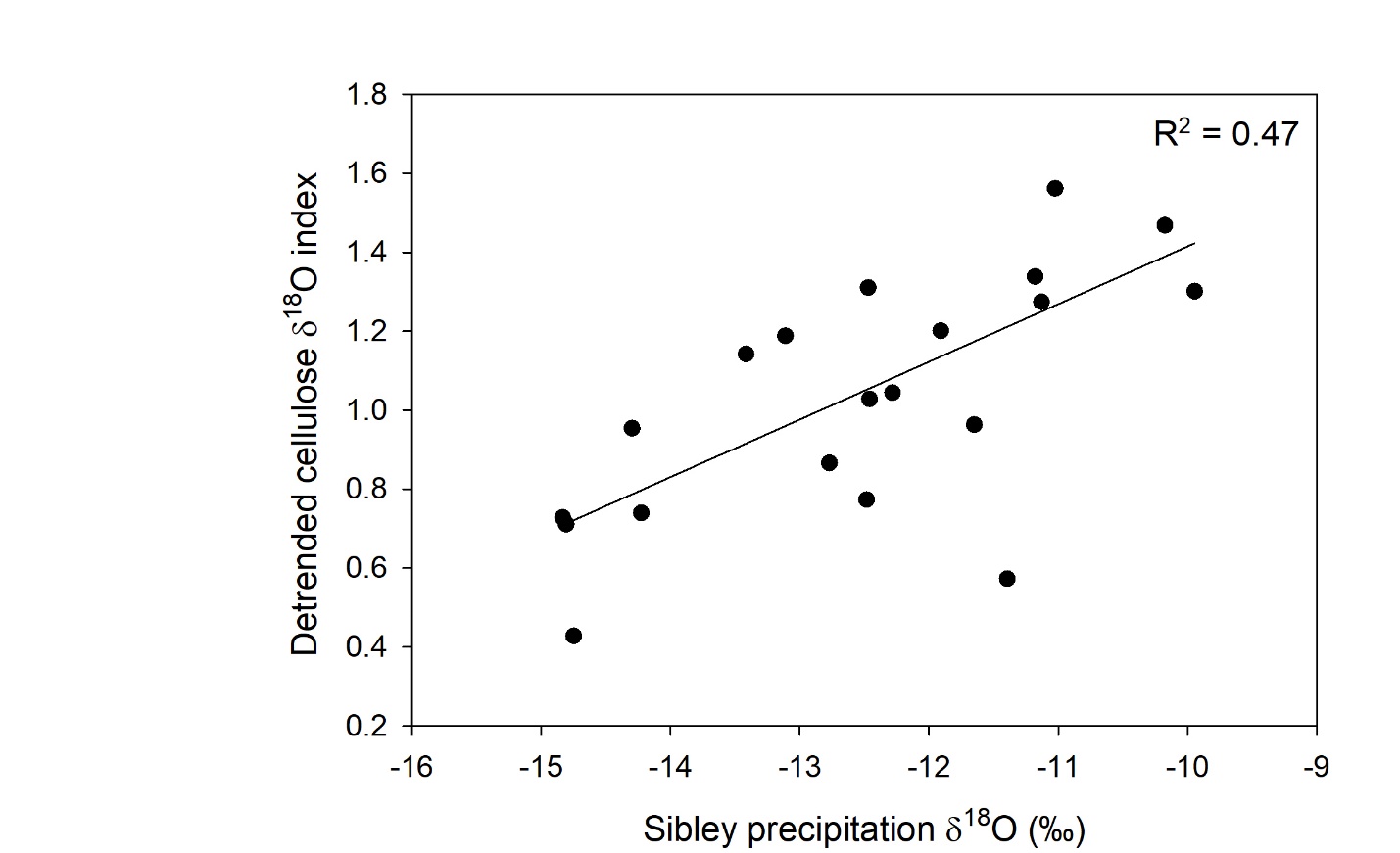


**Table 1.** The same format for results given in Table 2 but with regression models containing only current year Δ^13^C or δ^18^O data (i.e. without including variation in Δ^13^C or δ^18^O from the subsequent year). Geopotential height is abbreviated as HGT, see Methods for definitions of variables.

| Spatial scale | Variable | Model | Adjusted  R^2^ | | Model vs predictand correlation | P-value |
| --- | --- | --- | --- | --- | --- | --- |
| Local | Winter T_min_ | Δ^13^C | 0.16 | | -0.43 | **0.0061** |
| Local | Winter T_min_ | δ^18^O | 0.16 | | 0.43 | **0.0075** |
| Local | Winter T_min_ | Δ^13^C & δ^18^O | 0.34 | | 0.61 | **0.0002** |
| Local | Summer T_max_* | Δ^13^C | 0.00 | | -0.09 | 0.5991 |
| Local | Summer T_max_* | δ^18^O | 0.01 | | 0.20 | 0.2190 |
| Local | Summer T_max_* | Δ^13^C & δ^18^O | 0.00 | | 0.22 | 0.4053 |
| Regional | Near-lake Winter T_min_ | Δ^13^C | 0.21 | | -0.48 | **0.0018** |
| Regional | Near-lake Winter T_min_ | δ^18^O | 0.10 | | 0.35 | **0.026** |
| Regional | Near-lake Winter T_min_ | Δ^13^C & δ^18^O | 0.32 | | 0.60 | **0.0003** |
| Regional | Near-lake cloud cover | Δ^13^C | 0.00 | | 0.10 | 0.5257 |
| Regional | Near-lake cloud cover | δ^18^O | 0.08 | | -0.31 | 0.0500 |
| Regional | Near-lake cloud cover | Δ^13^C & δ^18^O | 0.06 | | 0.33 | 0.1100 |
| Regional | Peak ice cover | Δ^13^C | 0.27 | | -0.54 | **0.0003** |
| Regional | Peak ice cover | δ^18^O | 0.12 | | 0.37 | **0.0172** |
| Regional | Peak ice cover | Δ^13^C & δ^18^O | 0.41 | | 0.67 | **<0.0001** |
| Large-scale | HGT | Δ^13^C | 0.24 | | -0.51 | **0.0007** |
| Large-scale | HGT | δ^18^O | 0.00 | | 0.09 | 0.5793 |
| Large-scale | HGT | Δ^13^C & δ^18^O | 0.23 | | 0.52 | **0.0031** |
| Large-scale | Dipole index | Δ^13^C | 0.27 | | -0.54 | **0.0004** |
| Large-scale | Dipole index | δ^18^O | 0.01 | 0.18 | | 0.2561 |
| Large-scale | Dipole index | Δ^13^C & δ^18^O | 0.28 | 0.56 | | **0.0008** |

**Table 2.** Correlations between each stable isotope chronology and Michigan Division 1 Palmer Drought Severity Index (PDSI) values across the years 1976-2015. There were no significant relationships present between isotope chronologies and PDSI for individual months or for PDSI averaged across the spring (MJJ) and summer seasons (JJA) portions of the growing season that could potentially have influenced isotope signals during middlewood formation.

| Variable | Jan | Feb | Mar | Apr | May | Jun | Jul | Aug | Sep | Oct | Nov | Dec | MJJ | JJA |
| --- | --- | --- | --- | --- | --- | --- | --- | --- | --- | --- | --- | --- | --- | --- |
| δ^18^O | -0.16 | -0.10 | 0.01 | -0.11 | -0.12 | -0.20 | -0.21 | -0.16 | -0.16 | -0.08 | -0.06 | -0.09 | -0.19 | -0.21 |
| Δ^13^C | -0.13 | -0.18 | -0.29 | -0.22 | -0.21 | -0.16 | -0.01 | -0.05 | -0.06 | -0.07 | -0.13 | -0.14 | -0.13 | -0.08 |
